# Congenital Chagas Disease: Recommendations for Diagnosis, Treatment and Control of Newborns, Siblings and Pregnant Women

**DOI:** 10.1371/journal.pntd.0001250

**Published:** 2011-10-25

**Authors:** Yves Carlier, Faustino Torrico, Sergio Sosa-Estani, Graciela Russomando, Alejandro Luquetti, Hector Freilij, Pedro Albajar Vinas

**Affiliations:** 1 Laboratoire de Parasitologie, Faculté de Médecine, Université Libre de Bruxelles, Brussels, Belgium; 2 Facultad de Medicina, Universidad Mayor de San Simon, Cochabamba, Bolivia; 3 Centro Nacional de Diagnóstico e Investigación de Endemo-epidemias, Buenos Aires, Argentina; 4 Instituto de Investigaciones en Ciencias de la Salud, Universidad Nacional de Asunción, Rio de la Plata y Lagerenza, Asunción, Paraguay; 5 Laboratório de Pesquisa da doença de Chagas, Hospital das Clinicas, Universidade Federal de Goias, Goiania, Brazil; 6 Programa Nacional de Chagas (Ministerio de Salud Pública) y Servicio de Parasitología y Chagas (Hospital de Niños Ricardo Gutiérrez), Buenos Aires, Argentina; 7 Department of Control of Neglected Tropical Diseases, World Health Organization, Geneva, Switzerland; Institute of Tropical Medicine, Belgium

In May 2010, the sixty-third World Health Assembly adopted resolution WHA63.20 on the control and elimination of Chagas disease, highlighting the need “to promote the development of public health measures in disease-endemic and disease non-endemic countries, with special focus on endemic areas, for the early diagnosis of congenital transmission and management of cases” [1]. This article summarizes the recommendations of the Technical Group IVa on “Prevention and Control of Congenital Transmission and Case Management of Congenital Infections” of the World Health Organization's Programme on Control of Chagas disease (infection with *Trypanosoma cruzi*). The present recommendations derive from those obtained in the meetings listed in Box 1.

**Box 1. Meetings from Which the Present Recommendations Derive**

- Meeting ULB (Belgium)/UMSS (Bolivia)), Cochabamba, Bolivia, November 6–8, 2002: “Congenital Infection with *Trypanosoma cruzi*: From Mechanisms of Transmission to Strategies for Diagnosis And Control”, Carlier Y and Torico F, Revista da Sociedade Brasileira de Medicina Tropical 2003, 6: 767–771.
- Meeting PAHO/CLAP/ULB (Belgium)/IRD (France), Montevideo, Uruguay, June 24–25, 2004: “Congenital Chagas Disease: Its Epidemiology and Management”, http://www.paho.org/English/AD/DPC/CD/dch-chagas-congenita-2004.htm
- Meeting PAHO/CLAP/ULB (Belgium), Montevideo, Uruguay, May 17–18, 2007: “Information, Education and Communication in Congenital Chagas Disease”, http://www.paho.org/English/AD/DPC/CD/dch-congenita-iec-07.doc
- Meeting WHO, Geneva, Switzerland, July 4–6, 2007: “Revisiting Chagas Disease: From a Latin American Health Perspective to a Global Health Perspective”
- Meeting of the WHO TG IVa (congenital and paediatric Chagas disease), New Orleans, Louisiana, United States, December 11, 2008, satellite meeting to the ASTMH 57th annual meeting
- Meeting of the 6th European Congress of Tropical Medicine and International Health, Verona, Italy, September 6–10, 2009: “Chagas Disease in Europe”
- Meeting of WHO-HQ and the WHO regional office for Europe, Geneva, Switzerland, December 17–18, 2009: “Consultation on Chagas Disease in Europe”

## Preliminary Considerations on Congenital Transmission of *T. cruzi* Infection

Congenital transmission of *T. cruzi* infection is considered as such (i) when a neonate is born from an infectious mother (that is, a mother with positive serology or *T. cruzi* parasites circulating in the blood), and (ii) when *T. cruzi* parasites are identified at birth, or (iii) when *T. cruzi* parasites or specific antibodies not of maternal origin are detected after birth, and when previous transmission to infant by vectors and blood transfusion has been ruled out [2], [3].

Congenital transmission occurs in areas where the disease is endemic as well as in areas where vector transmission has been interrupted, in areas where the disease is non-endemic, and from one generation to another. This pattern of transmission facilitates uncontrolled spread of the parasite infection for long periods of time [2], [3].

According to epidemiological data from Latin America, the estimated number of cases of congenital *T. cruzi* infection is >15,000 per year [4]. The incidence of congenital cases in non-endemic areas is not known, although several reports attest to its occurrence [5], [6].

Although some congenital cases can present non-specific symptoms as is seen in congenital infections with *Toxoplasma gondii*, rubella virus, cytomegalovirus, and herpes simplex virus (commonly identified in the acronym TORCH), most cases are asymptomatic. This warrants a control strategy of mandatory screening for congenital infection based on laboratory diagnosis [2], [3], [7], [8], [9].

Congenital *T. cruzi* infection is an acute infection in newborns that should be treated with anti-parasitic therapy. Left untreated, the infection can progress to chronic Chagas disease later in life.

## Prevention of Congenital Transmission

For pregnant women who are already infected with *T. cruzi*, there is no specific or direct means of preventing congenital infection. Since the teratogenic risks of the available medicines (benznidazole and nifurtimox) are not well known and the risk of adverse reactions is high in adults, anti-parasitic treatment is not recommended during pregnancy.

For women who are not pregnant, prevention of congenital transmission is possible by (i) treating infected women aged ≤15 years and infected women of fertile age who are living or have lived in disease-endemic areas [10] (outside lactation periods to avoid interruption of lactation as a result of possible adverse reactions to medicines [11]); and (ii) control of vectors and blood transmission in disease-endemic areas to reduce the risk of infection and the reservoir of infected women [12].

## Antenatal Screening

Screening should be carried out during pregnancy to detect mothers who are infectious and at risk of transmitting infection to their foetuses. There is no way to identify, in advance, those mothers who will transmit the infection to their fetuses.

Serological testing is recommended for pregnant women (i) who are living in disease-endemic areas, (ii) who are living in disease non-endemic areas and have occasionally received blood transfusion in disease-endemic areas, and (iii) who are living in disease non-endemic areas and are born or have lived previously in disease-endemic areas or whose mothers were born in such areas.

## Diagnostic Methods

### Detection of Infection during Pregnancy

Detection of infection during pregnancy can be performed using two conventional serological tests: indirect immunofluorescence assay (IFA); enzyme-linked immunosorbent assay (ELISA). These diagnostic tests are generally available at low cost in primary health care facilities; however, they have to be performed in a laboratory and the results are not immediately available [13], [14].

Rapid diagnostic methods (such as inmunochromatographic, immunodot, and immunofiltration tests) are necessary for pregnant women entering maternity facilities for delivery without previous serodiagnosis, or at primary health care facilities in rural endemic areas. These screening tests are quickly performed, but they have not been validated at large scale and need confirmation with standard serological tests [15], [16].

### Detection of Congenital Infection in Neonates

Detection of congenital infection in neonates can be performed by detecting living parasites in the blood (umbilical cord blood or venous blood of the newborn). Parasitological techniques that concentrate parasites by centrifugation can be performed using capillary tubes (microhematocrit test; investigation of parasites by microscopic examination of blood buffy coat can be done directly into the tube, avoiding its cutting, which was susceptible to contaminating the examiner), or Eppendorf tubes (microstrout). These tests offer rapid and definitive diagnosis allowing for rapid initiation of treatment. However, they require skilled personnel and assured quality control, which may not be available in primary health care facilities in rural endemic areas [2], [3], [17].

Polymerase chain reaction (PCR) is under evaluation and has not been validated yet for the diagnosis of congenital infection, although it might improve its early detection [9], [18], [19]. It is not available at primary health care facilities in endemic areas and has to be reserved for specialised centres.

### Detection of Congenital Infection in Infants Aged >8 Months

Detection of congenital infection in infants aged >8 months (when maternal antibodies have disappeared) can be performed by detecting *T. cruzi*–specific antibodies using serological tests, as for mothers. These tests are available at primary health care facilities. However, their performance in infants aged >8 months of age delays diagnosis and treatment. A negative serological result in infants below 8 months of age indicates an absence of congenital infection.

Detection of blood parasites at any time after birth or a positive *T. cruzi*–specific serology in infants aged >8 months are the gold standards for diagnosis of congenital Chagas disease (when previous transmission by vectors and blood transfusion has been ruled out, see above).

Health systems should evaluate the strategy that facilitates the earliest possible diagnosis of congenital infection.

## Treatment of Neonates and Infants

Cases of congenital *T. cruzi* infection should be treated as soon as the diagnosis has been confirmed.

Although randomised comparative clinical trials have not been carried out, the experience of expert clinical groups in treating congenital *T. cruzi* infection indicates that (i) both benznidazole and nifurtimox can be used to treat congenital cases; (ii) the recommended dose of benznidazole in infants, as in adults, is 5–7 mg/kg per day; doses of benznidazole up to 10 mg/kg per day can be used in neonates and infants aged <1 year [12]; the recommended doses of nifurtimox in neonates and infants are 10–15 mg/kg per day; (iii) such doses can be administered orally in one dose in low-weight neonates or, preferably, in divided doses of two to three sub-doses; precautions should to be taken to obtain appropriate dosage of active drug, since the currently available tablets have to be crushed and used as a suspension; (iv) the recommended duration of treatment is 60 days and should not be <30 days; (v) benznidazole, which is manufactured by Laboratório Farmacêutico do Estado de Pernambuco (LAPEFE, Brazil), is available in tablets of 100 mg through “Masters” (Davie, Florida, United States; Elstree, Hertfordshire, United Kingdom), the World Health Organization (WHO), and the Pan American Health Organization (PAHO). To facilitate the preparation of paediatric suspensions, benznidazole should also be available in dispersal tablets of 12.5 mg. Nifurtimox is manufactured by Bayer and is available in tablets of 120 mg through WHO and PAHO.

Treatment is generally successful and without the adverse reactions seen in adults if administered within the first year of life [20].

## Care of Infected Mothers and Their Other Infected Children

The clinical forms of maternal *T. cruzi* infection (particularly the cardiac and digestive forms) require further evaluation [12]. Since etiological treatment of infected women is not recommended during pregnancy, treatment should be considered after delivery and breastfeeding.

It is recommended that infected mothers do not donate blood. Medical evaluation with electrocardiography should be performed regularly to follow the clinical evolution of the disease.

Chagas disease should be systematically investigated in siblings and relatives of infected mothers (serological investigation), and positive cases should be clinically evaluated and treated accordingly.
